# Palliative care in glioblastoma patients: a systematic review

**DOI:** 10.1590/1806-9282.2024S122

**Published:** 2024-06-07

**Authors:** Ligia Henriques Coronatto, Cleiton Formentin

**Affiliations:** 1 Universidade Federal de São Paulo, Neuro-Oncology Sector, Department of Neurosurgery – São Paulo (SP), Brazil.; 2 Hospital Sírio Libanês – São Paulo (SP), Brazil; 3 Universidade Estadual de Campinas, Department of Neurology, Neurosurgery Discipline – Campinas (SP), Brazil.

## INTRODUCTION

Glioblastoma (GBM) is a type of brain tumor that belongs to the subgroup of gliomas. It is the most common malignant primary brain tumor in adults^1,2^. The average age at diagnosis is around 65 years, and the incidence rate is approximately 3.23 cases per 100,000 people³. The average life expectancy is 10–15 months, and the survival rate is estimated at 2 years in 25% of patients^3^.

The standard treatment for newly diagnosed GBM became the Stupp protocol after its publication in 2005, leading to significant improvements in survival rates^4^. However, it's important to note that GBM remains incurable despite multimodal treatment approaches involving surgery, radiotherapy (RT), and chemotherapy (CT). This aggressive form of brain cancer can have a profound impact on various aspects of a person's life, resulting in a range of progressive and often concurrent symptoms such as headaches, hemiparesis, cognitive disorders, personality changes, and language difficulties. The complexity of these symptoms and problems can negatively affect the health-related quality of life (HRQoL) not only of patients but also of their relatives^3^.

The fact that GBM patients have a progressive brain disease can seriously interfere with their ability to make their own treatment decisions. It is worth noting that soon after diagnosis, approximately half of glioma patients already encounter challenges in making treatment decisions, and this percentage tends to increase as the disease progresses toward its terminal stages^1^. Therefore, the importance of initiating advance care planning (ACP) and introducing GBM patients to palliative care (PC) early in the treatment process cannot be overstated^5–9^. There is evidence to suggest that early PC, coupled with structured ACP, with a focus on topics such as timely identification and treatment of disease-specific symptoms, can enhance the HRQoL for GBM patients and improve symptom management^7^.

The purpose of this systematic review is to evaluate the quality of life of GBM patients, regardless of the treatment performed, with a specific focus on PC and end-of-life care.

This review will assess various dimensions of quality of life, including physical, psychological, and social well-being.

## METHODS

We conducted a literature search encompassing the past 10 years, from October 2012 to October 2022, using terms such as "glioblastoma," "palliative care," "terminal care," "end-of-life care," "hospice," and "quality of life." The search was performed in multiple databases, including Cochrane, PubMed, LILACS, the Virtual Health Library, and EMBASE, resulting in the identification of 4,985 articles. From this pool, we selected only complete works published in the English language that were freely accessible, including completed clinical trials, literature reviews, systematic literature reviews, case-control studies, cross-sectional studies, and cohorts. No meta-analysis was found. We excluded case reports, case series, letters, and published abstracts. Duplicate titles were removed, leaving a total of 24 articles that met all the inclusion criteria.

The primary objective of this review is to evaluate the quality of life of GBM patients, focusing on PC and end-of-life care. As secondary objectives, we evaluated the communication of prognosis to patients and family members/caregivers, ACP, and care for patients, family members, and caregivers at the end of life.

## RESULTS

### Quality of life

The vast majority of studies do not use objective tools to assess HRQoL in GBM patients. Studies generally consider the following aspects to assess quality of life: performance status, pain, dyspnea, headache, swallowing disturbances, dysarthria, drowsiness, behavioral changes, depression, anxiety, spirituality, motor changes, seizures, visual changes, incoordination, language changes, sphincter changes, and being independent, among others. The heterogeneity of the studies becomes an important limitation for quality-of-life assessment^10–12^.

### Prognosis communication

Prognosis communication should start early in the trajectory of serious illnesses and be embedded in broader conversations about patients' priorities. However, it is observed that these conversations and PC occur near the end of life. Clear and frank conversations regarding the prognosis of GBM patients are associated with more goal-consistent care, a better quality of life, and better family coping^13^.

In all cancer types, discussions about prognosis are difficult for patients, caregivers, and oncologists, but are particularly challenging in the GBM setting due to cognitive and functional decline. In a prospective study of patients with malignant glioma and their caregivers, only 40% of patients had a full understanding of their prognosis, while 69% of caregivers had full knowledge of the patient's prognosis^14^. Physicians need to communicate more clearly about aspects of prognosis to help patients and caregivers understand the importance of ACP. Studies have not yet clarified the ideal time to communicate the prognosis to GBM patients^14^.

### Advance care planning

Progressive cognitive decline in GBM patients can seriously interfere with the patients' ability to make treatment or care decisions. It is important to involve patients with glioma early in the disease trajectory in making decisions about future care, including treatment and end-of-life care. One way to achieve this is with ACP. Just as the optimal time for communicating the prognosis to GBM patients is not yet defined, there is also no universally agreed-upon timing for introducing ACP^7^.

**Figure 1:**
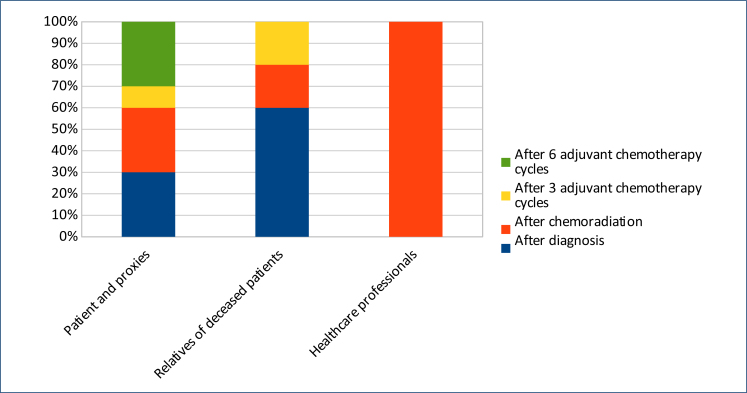
Preference for the moment of the disease trajectory in which the advance care planning program should be implemented (adapted from "Fritz et al.^7^").

Healthcare professionals unanimously decided that the ideal time to offer ACP to patients would be after chemoradiation^7^. The main reason for this decision is that patients are still competent, have no cognitive decline early in the disease trajectory, and are therefore usually able to communicate their desires. However, family members believe that the best time is once the diagnosis is made. For patients, the best moment is divided mainly between the time of diagnosis, after chemoradiation and after six cycles of adjuvant CT (Figure 1)^7^.

In patients with malignancies, advanced and systematic care planning has been associated with numerous benefits, including less aggressive end-of-life care, improved quality of life for both patients and caregivers, earlier involvement in PC, reduced psychological distress, care alignment with patient preferences, cost-effective care, and potentially improved survival. However, research in the context of GBM is limited. Much of the existing data encompass a broader population of malignant brain tumors, making interpretation challenging due to variations in prognosis among different tumor types^15^.

### End-of-life care

For patients with incurable cancer, it is critical to provide end-of-life medical and psychosocial care that minimizes symptom burden and optimizes quality of life. At the end of life, GBM patients suffer a wide variety of neurological symptoms, including headaches, incontinence, and seizures, as well as non-neurological symptoms such as acute infection or pulmonary embolism. In one study, 20% of patients with malignant brain tumors were referred to PC 1 week before death, and 59% were referred within 30 days before death^16^. There is still no consensus on when the final stage of life begins in patients with glioma.

**Figure 2:**
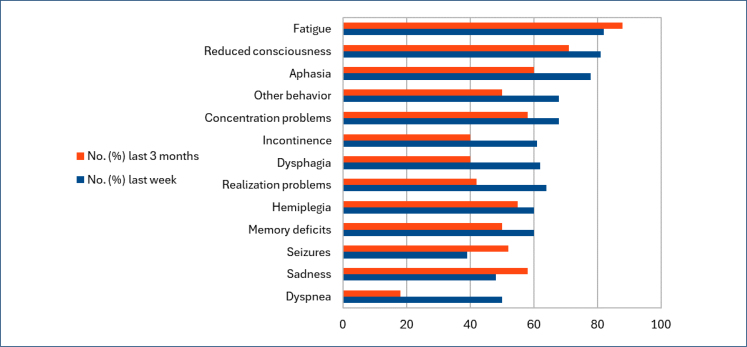
Symptoms reported during the last three months of life and the last week of life (adapted from "Flechl et al.^17^").

Fatigue was the most common symptom in GBM patients during the end of life, followed by a decreased level of consciousness and aphasia (Figure 2). Only 20% of patients remained fully independent in their mobility, reflecting the high need for care for GBM patients during the final stage of life^17^.

A study demonstrated that CT frequently used among GBM patients close to death does not benefit survival and may even worsen these patients' quality of life^18^. It is reported that a quarter of patients do not die with dignity, which may be due to several factors, including inadequate training of the physicians and caregivers providing end-of-life care, inadequate healthcare environments in the terminal phase, and the patient's inability to communicate effectively^19^.

**Figure 3:**
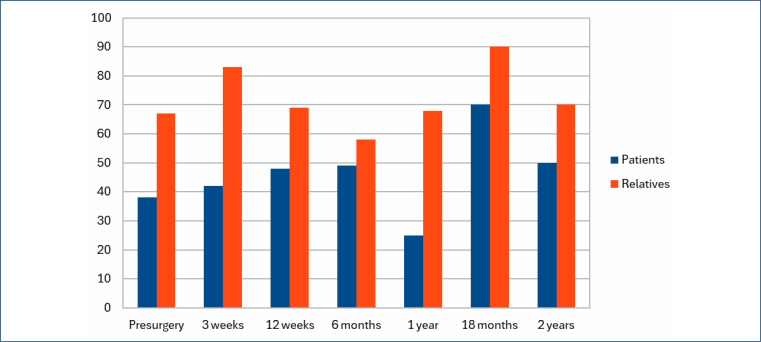
Percentage of patients and family members experiencing anxiety symptoms (hospital anxiety and depression scale ≥8) at different time periods (adapted from "Ståhl et al.^20^").

One study confirmed that HRQoL and emotional well-being are affected not only in patients but also in family members.^20^ In fact, relatives are more vulnerable before surgery in terms of mental health and emotional well-being than the patients themselves. Family members scored worse on items covering mental HRQoL and reported more frequent symptoms of anxiety and depression than GBM patients themselves (Figure 3)^20,21^.

## DISCUSSION

Quality of life is a complex entity that originates from the interaction between a person's values and expectations and their actual experience. It is defined as a multidimensional concept consisting of social, psychological, and physical phenomena. Fatigue, poor sleep quality, inability to concentrate, depression, financial burden, and impaired personal and social relationships significantly impact the quality of life for GBM survivors. Moreover, various domains of cognition, including sustained attention, long-term memory, mental flexibility, and executive functions, are notably impaired, thereby affecting these patients' personal, social, and professional lives. These impairments in cognitive functions are multifactorial, resulting from the tumor itself, the surgery, radiation therapy, and the effects of CT^22^. Cognitive deterioration is considered the main indicator of poor disease progression after treatment^23^.

There was also an apparent correlation between tumor progression and the deterioration of HRQoL scores. This indicates that progression-free survival is not only a surrogate marker for survival but also for quality of life. Patients with persistent and progressive decline in HRQoL shortly after surgery may represent a subgroup with a worse prognosis, despite active treatment. Independent predictors of better quality of survival at 1 year after diagnosis were high preoperative Karnofsky score and gross total resection, while preoperative cognitive symptoms were predictive of poorer quality of survival^24^.

Many patients with high-grade glioma receive less PC than other cancer patients, despite the high symptom burden. An optimal model for integrating early PC into the treatment of gliomas has not been established. Patient screening to initiate PC is variable. The use of a PC screening tool can facilitate early referral to PC and improve patient outcomes in symptom management and quality of life^25^.

Standard PC assessments have identified the primary burdens faced by GBM patients as psychosocial issues and increased dependence on care. These assessments should be supplemented with specific questions about neuropsychiatric symptoms and family caregiver burden, as both factors are of utmost importance and significantly influence the well-being of patients and their caregivers^26^. There is a pressing need to enhance the quality of life for both patients and caregivers, as they often have multiple supportive care needs. To address these needs effectively, a multidisciplinary approach focusing on ACP should be initiated as soon as the GBM diagnosis is established. This approach is considered the most suitable way to provide comprehensive treatment along with PC, which can improve the long-term quality of life for these patients and prevent unnecessary suffering at the end of life^26^.

## CONCLUSION

There are still relatively few studies that specifically address the quality of life in GBM patients, and most of these studies tend to assess quality of life subjectively. This underscores the importance of developing a standardized scale for assessing the quality of life in future studies involving these patients. Early PC is crucial for GBM patients and their families, as it helps preserve quality of life despite aggressive tumor progression even with current therapies.
